# ERAS guidelines for hip and knee replacement – need for reanalysis of evidence and recommendations?

**DOI:** 10.1080/17453674.2020.1728920

**Published:** 2020-02-20

**Authors:** Henrik Kehlet, Stavros G Memtsoudis

**Affiliations:** a Rigshospitalet, Section of Surgical Pathophysiology, Copenhagen, Denmark;; b Department of Anesthesiology, Critical Care, and Pain Management, Hospital for Special Surgery, New York, USA Department of Healthcare Policy and Research, Weill Cornell Medical College, New York, USA Department of Anesthesiology, Perioperative Medicine and Intensive Care Medicine, Paracelsus Medical University, Salzburg, Austria

In a laudable effort, 8 multidisciplinary authors produced a consensus statement for perioperative care in hip and knee replacement (THA/TKA) recently published in *Acta Orthopaedica*. The primary goal was to evaluate “efficacy of individual items of the perioperative treatment pathway to expedite the achievement of discharge criteria” (Wainwright et al. 2020). In this context, the consensus group addressed 17 topics that were assessed based on a proposed systematic review of the literature from January 1966 to October 2018, with the purpose to reflect evidence at the time of writing in January 2019.

While this type of effort is important in order to provide the wider perioperative and anesthesia community with guidance, its publication comes with a great deal of responsibility, as practitioners will use this information to model their practice. Therefore, clinicians need to not only read the conclusions, but be aware of the sources of evidence they are based on. In this context, it must be acknowledged that this consensus document provides an enormous amount of valuable information, however, a number of points should be considered. Specifically, a closer look at the 17 considered recommendations may lead to questions regarding the search methodology as well as the concept of including so many factors, which may be of limited importance in fulfilling the primary aim of the consensus report, namely the earlier achievement of discharge criteria. It must be asked if the inclusion of this large number of interventions is indeed feasible and/or justifiable for such a project. This concern is based on the fact that the information considered was to a large extent not procedure specific nor was it – in many cases – derived from studies conducted during a time that is compatible with documented modern ERAS clinical practice in fast-track THA/TKA. Another factor to consider is that the group’s focus was heavily weighted towards specific clinical studies, thus frequently ignoring information gained from other literature sources, such as population-based investigations, which when considered can provide valuable information.

As to the proposed recommendations, preadmission patient optimization regarding smoking and alcohol, although arguably appropriate, is not based on hard scientific, procedure-specific data. Indeed, much of the information is based on transferable evidence from other procedures and not supported from the few fully implemented THA/TKA ERAS data. Preoperative information is obviously to be implemented according to common practice in most countries, but is not a unique procedure-specific item on the specified discharge criteria or documented to enhance discharge in THA/TKA. Preoperative fasting has been emphasized repeatedly in all previous ERAS guidelines. Further, it is a well-established fact that oral intake does not have to be withheld for long. However, there is no procedure-specific evidence from THA/TKA where the patient in modern practice care is admitted on the morning before surgery. The same applies to preoperative carbohydrate treatment, which is not recommended as an essential or routine intervention. To include pre-anesthetic anxiolytic medication is neither procedure-specific nor evidence-based and is obviously not necessary in other types of modern surgery. The recommendations for general vs. neuraxial anesthesia are discussed rather superficially and lead to the conclusion that either technique can be used. Here, the recommendation conflicts with recent (and previous) data suggesting that neuraxial approaches may be preferable (Memtsoudis et al. 2019a). However, the latter conclusion is one that stems from the evaluation of a much broader literature base, including population data that considers complication risk. The topics of spinal opioids and epidurals get to the right conclusion not to be recommended, although the literature basis for this suggestion is superficially presented with regard to side effects. Additionally, the important and large topic of pain management, which is crucial to achieving early mobilization and shortening discharge time (the primary aim for the guideline) is mostly based on old references in which a length of stay (LOS) > 2 days was prevalent in most studies. This fact and the rather limited literature review does therefore not seem to appropriately support the current clinical practice. The same applies to the multimodal analgesia section which also suffers from the surprising lack of discussion of the use of preoperative high-dose steroids (2 references mentioned, of which 1 is not with high-dose) despite the availability of several systematic reviews and RCT’s with rather promising results published before manuscript submission. Further, there is a lack of references to critical systematic reviews on pain management (Hojer Karlsen et al. 2015, Karlsen et al. 2017) and an absence of discussion of whether these data apply to a fast-track THA/TKA setup.

Next, the section of surgical traditions that potentially impair early recovery such a urinary catheterization, use of tourniquets, drains, etc. is appropriately discussed although again with limited references. This is important as these care principles are still widely used and should be eliminated – as quoted – to achieve a shorter LOS and recovery time. The section on normothermia is not based on THA/TKA data and represents standard of care in modern surgical practice. The same applies to antibiotic prophylactic practice which is irrelevant for achieving early discharge criteria, but obviously important for post-discharge outcome. The issue of anti-thrombotic prophylaxis, which presently is controversial and ranges from weeks of prophylaxis with expensive and potentially side-effect laden modern potent anticoagulants vs. the simple use of Aspirin, falls into a similar category. Importantly, the anti-thrombotic prophylaxis discussion is turning away from the primary issue of the guideline to shorten the time before discharge. The recommendation for early return to normal diet is – as mentioned – a component of all ERAS pathways, but specifically of limited relevance to THA and TKA procedures, which increasingly can be performed on an outpatient or 1-day basis. The section on continuous improvement and audit is obvious and important, but is again not procedure-specific with regards to recommendations for an optimal THA/TKA ERAS program.

Although, the recommended “interventions” have 17 strong-grade recommendations, only the ones suggesting the use of local infiltration analgesia in TKA, tranexamic acid, multimodal opioid-sparing analgesia and early mobilization are “active” components. In contrast, most of the other 17 components are not based on new scientific data to show that they may lead to a reduced LOS from previously 6–8 days to less than 2 days, or even to be performed on an outpatient basis.

Finally, busy clinicians may have a hard time interpreting the many guidelines on perioperative interventions in THA/TKA. For example, the recently published US guidelines (available as Epub in June/July 2018, but in print in March 2019) (Soffin et al. 2019a, b) are quite different from the present THA/TKA recommendations (Wainwright et al. 2020), although the former also suffer from the lack of a critical literature update (Kehlet and Joshi 2019).

In summary, despite the major and laudable efforts made by the multidisciplinary team when putting together this important information, there is still room for improvement with a more critical reanalysis of the data. The future discussion should focus on which components of care are most important in order to achieve a length of stay between 0 and 2 days, including a more important evaluation of the pathophysiology of recovery in general and in THA and TKA patients with certain characteristics in particular (high-pain responders, high inflammatory responders, psychosocial factors, etc.) (Memtsoudis et al. 2019b, Wainwright and Kehlet 2019). In addition, since major progress has been achieved with ERAS programs in THA and TKA, as evidenced by the reported shortening of LOS and several types of complications, the major questions that remain to be answered include the issue of post-discharge functional recovery, which to date is addressed by a paucity of literature (but apparently was not the focus of the article). However, post-discharge recovery is per definition part of the concept of “enhanced recovery after surgery”. In this context, there is not only a lack of good scientific data, but a need for updated guidelines for post-discharge functional recovery (Wainwright and Kehlet 2019).

Hopefully, future procedure-specific ERAS guidelines will be divided more specifically into items that constitute transferable evidence/routine perioperative care in modern surgery vs. well-documented interventions to achieve an improved and fully implemented procedure-specific ERAS program to enhance total recovery, shorten LOS, and decrease complications (Kehlet and Joshi 2019). This would allow groups of experts to focus their analytic efforts and discussions on procedure specific issues and address the many challenges that still remain unaddressed. Finally, ERAS recommendations may benefit from a clearer separation of components that are necessary for early recovery and the reduction of LOS vs. those that need to be considered for post-discharge functional recovery.
